# Isolation and identification of a novel, lipase-producing bacterium, *Pseudomnas aeruginosa* KM110

**Published:** 2011-06

**Authors:** E Mobarak-Qamsari, R Kasra-Kermanshahi, Z Moosavi-nejad

**Affiliations:** Department of Biology, Alzahra University, Vanak, Tehran, Iran

**Keywords:** Lipase, *Pseudomonas aeruginosa*, Stability

## Abstract

**Background and Objectives:**

Lipases are particularly important due to the fact that they specifically hydrolyze acyl glycerol, oils and greases, which is of great interest for different industrial applications.

**Materialst and Methods:**

In this study, several lipase-producing bacteria were isolated from wastewater of an oil processing plant. The strain possessing the highest lipase activity was identified both biochemically and sequencing of 16S rRNA gene. Then we increase lipase activity by improving conditions of production medium. Also, lipase from this strain was preliminarily characterized for use in industrial application.

**Results:**

The 16S rRNA sequensing revealed it as a new strain of *Pseudomonas aeruginosa* and the type strain was KM110. An overall 3-fold enhanced lipase production (0.76 U mL^−1^) was achieved after improving conditions of production medium. The olive oil and peptone was found to be the most suitable substrate for maximum enzyme production. Also the enzyme exhibited maximum lipolytic activity at 45°C where it was also stably maintained. At pH 8.0, the lipase had the highest stability but no activity. It was active over a pH range of 7.0–10.0. The lipase activity was inhibited by Zn^2+^ & Cu^2+^ (32 and 27%, respectively) at 1mM. The enzyme lost 29% of its initial activity in 1.0% SDS concentration, whereas, Triton X-100, Tween-80 & DMSO did not significantly inhibit lipase activity.

**Conclusions:**

Based on the findings of present study, lipase of *P. aeruginosa* KM110 is a potential alkaline lipase and a candidate for industrial applications such as detergent, leather and fine chemical industries.

## INTRODUCTION

Many attempts have been made to isolate lipase producing microorganisms since this enzyme is used in numerous biotechnological processes including food, leather, cosmetic, detergents and pharmaceutical industries and industrial wastes management (1). Lipases (triacylglycerol acylhydrolases, EC 3.1.1.3) catalyze the hydrolysis and the synthesis of esters formed from glycerol and long- chain fatty acids. Lipases occur widely in nature, but only microbial lipases are commercially significant. Microbial lipases are high in demand due to their specificity of reaction, stereo specificity and less energy consumption than conventional methods (2). Many microorganisms such as bacteria, yeast and fungi are known to secret lipases. Lipase-producing microorganisms have been found in diverse habitats such as industrial wastes, vegetable oil processing factories, dairies, soil contaminated with oil, etc (3). The oily environment (oil mill effluent) may provide a good environment for isolation of lipase producing microorganisms. Bacterial lipases are mostly extracellular and are greatly influenced by nutritional and physico- chemical factors, such as temperature, pH, nitrogen and carbon sources, inorganic salts, agitation and dissolved oxygen concentration (4). Among bacterial lipases, attention has usually been focused on particular classes of enzymes such as the lipases from the genus *Pseudomonas*, which are especially interesting for biotechnology. The literature reports different species of *Pseudomonas* from diverse environments producing lipase (5). The most important stage in a biological process is optimization to improve and increase the efficiency of the process without increasing the cost

The purpose of the present study is to isolate new bacteria from wastewater of an oil processing plant located in Tehran and increase its lipase activity by improving conditions of the production medium containing nitrogen and carbon sources. Also, Lipase from this strain was preliminarily characterized for use in industrial application.

## MATERIALS AND METHODS

**Screening of lipolytic bacteria**. Lipase producing microbial cultures were isolated from wastewater of an oil processing plant located in Tehran, and enriched by periodic subculturing of samples in Nutrient Broth (NB) media containing 20% (v/v) and 40% (v/v) wastewater in successive. The composition of NB medium is (per liter) 5g peptone and 3g yeast extract. The pH of the medium was adjusted to 7 with 0.1M NaOH.The isolation process was performed by serial dilution of samples on tributyrin agar plates. The composition of tributyrin agar medium is (per liter) 5 g peptone, 3 g yeast extract, 10ml tributyrin and 15 g agar. Culture plates were incubated at 30°C. Colonies showing clear zones around them were picked out, purified on tributyrin agar plates and transferred to agar slants (6). Isolates having clearing zone were grown in the liquid culture and the level of lipase production was determined from the cell free culture supernatant fluid. Characterization and identification of the isolate with higher lipolytic activity was carried out both biochemically and by 16s r RNA sequencing.

**Enzyme production**. The composition of production medium used in this study was: (%w/v) pepton 0.2; NH_4_H_2_PO4 0.1; NaCl 0.25; MgSO_4_ 7H_2_O 0.04; CaCl_2_.2H_2_O 0.04; olive oil 2.0 (v/v); pH 7.0; 1–2 drops Tween 80 as emulsifier. Overnight cultures were suspended in 5ml of sterile deionised water and used as the inoculum for pre culture to obtain an initial cell density to adjust the turbidity of 0.5 McFarland standard. Submerged microbial cultures were incubated in 500 ml Erlenmeyer flasks containing 100 ml of liquid medium on a rotary shaker (150 rpm) and incubated at 30°C. After 24 hours of incubation, the culture was centrifuged at 10,000 rpm for 20 min at 4°C and the cell free culture supernatant fluid was used as the sources of extracellular enzyme.

**Assay of lipase activity**. Lipase activity was deter-mined spectrophotometrically at 30°C using p-nitrophenol palmitate (pNPP) as substrate. The reaction mixture was composed of 700 µl *p*NPP solution and 300 µl of lipase solution. The *p*NPP solution was prepared by adding the solution A (0.001 g *p*NPP in 1ml isopropanal) into solution B (0.01 g gum arabic, 0.02 g Sodium deoxycholate, 50 µl Triton X-100 and 9 ml of 50 mM Tris-HCl buffer, pH 8) with stirring until all was dissolved. Then the absorbance measured at 410 nm for the first 2 min of reaction. One unit (1U) was defined as that amount of enzyme that liberated 1µmol of *p*NPP per minute (ɛ:1500*l*/mol cm) under the test conditions (7).

**Effects of culture variable on lipase production**. To investigate lipase production, olive oil was replaced by other carbon sources such as glucose and tributyrin. Each of substances (1% w/v) was used as sole carbon source. The effect of nitrogen sources on the lipase production was analyzed by supplementing production medium with different nitrogen sources (0.2% w/v) like peptone, yeast extract, ammonium dihydrogen phosphate and enzyme activity was assayed. Investigation of effect of different carbon and nitrogen source on lipase activity of *Pseudomonas aeruginosa* KM110 was done at pH: 7.0, 30°C and 150 rpm throughout 24 h of cultivations.

**Effect of pH and temperature on lipase activity and stability**. The crude enzyme used for assay was the culture broth after separation of cells and particles. The enzyme was normally stored at 4°C until used. The optimal temperature for activity was determined at different temperatures (30–70°C), at pH 8.0 for 10 min. For determination of temperature stability, the reaction mixtures containing the enzyme in 50mM Tris–HCl buffer (pH 8.0) was incubated at different temperatures (37, 45, 50, 55, 65 and 70°C) for 3 h and immediately cooled. Residual enzyme activity was measured under standard enzyme test conditions. Optimal pH was determined at 30°C in buffer solutions of pH values ranging from 5 to 11 (0.05 M citrate-phosphate pH 5-7; 0.05 M Tris–HCl pH 8-9; 0; 0.05 M Glysin – NaOH pH 11). The effect of pH on enzyme stability was analyzed by the spectrophotometric assay after pre-incubation of 300 µl of enzyme solution for 1 h at 30°C, in 700 µl of the above mentioned buffer solutions (pH 5–11). Enzymatic activity was measured according to a standard protocol with *p*NPP as the substrate.

**Effects of different ions & detergents on lipase activity**. As reported from studies on other microbial lipases, a concentration as low as 1 mM of some metal ions can affect the enzyme activity. Thus, the effect of several ions (Fe2+,,Na+, Ni+, Li+, Co2+, K+, Zn2+, Hg2+, Cu2+, Mn2+, Ca2+, Mg2+) on this *P. aeruginosa* lipase was determined. The enzyme solution was stored for 1 h at 30°C in the presence of 1 mM of various ions (as chloride salts) prior to the colorimetric assay for remaining lipase activity. In the case of chemical detergents, activity remaining was determined after 1 h of storage of enzyme solution at 30°C in the presence of various chemical detergents (SDS, DMSO (dimethyl sulfoxide), Tween 80 and Triton X-100) at 1% concentration. Activity was measured by the spectrophotometric assay after incubation time. Remaining enzymatic activity was determined by a standard method with *p*NPP. Final enzyme activity was calculated relative to control activity (a parallel enzyme reaction without additions).

**Taxonomic characterization of isolated bacteria**. The isolate was identified via 16S rRNA sequences. Genomic DNA of *Pseudomonas aeruginosa* KM110 was extracted from bacterial colonies by set buffer method. The 16S rRNA gene from the genomic DNA was amplified by PCR using the following forward and reverse primers of 16S rRNA, f (5′-AGAGTTTGATCMTGGCTCAG-3′) and r (5′- TACGGYTACCTTGTTACGAC-3′).PCRwas performed in a Thermocycler (TECHNE) using a *Taq* polymerase (Cinnagen, Iran). The PCR program comprised initial denaturation at 96°C for 4 min, followed by 35 cycles each of 94°C for 1 min, 61°C for 30 s, 72°C for 50 s; 72°C for 4 min; and incubation at 4°C for 10 min. PCR products were purified with DNA extraction kit (Bioneer South Korea). Both strands of the PCR product were sequenced by dideoxy chain termination method. The 16S rRNA gene sequence of the KM110 was compared with those in the NCBI/EZtaxon/ Ribosomal Database Project (RPD)/ EMBL nucleotide sequence databases by using the BLAST (blastn) program http://www.ncbi.nlm.nih.gov/BLATS/), and all of the sequences were aligned using the Clustal W program (8). A phylogenetic tree and neighbor-joining phylogeny were constructed by using the MEGA soft ware package version 4.0 (9) and bootstrapping was used to estimate the reliability of the phylogenetic reconstructions (1,000 replicates).

## RESULTS AND DISCUSSION

**Screening and identification of lipolytic bacteria**. The almost complete 16S rRNA gene was sequenced and the (1406 bp) analysis clearly demonstrated that strain KM110 was a member of the genus *Pseudomonas* and exhibited maximum similarity with the 16S rRNA sequence of *Pseudomonas aeruginosa* LMG 1242T(Z76651) (98.94% sequence similarity). This sequence data has been submitted to the DDBJ/EMBL/GenBank databases under accession No. HQ730879.

**Fig. 1:**
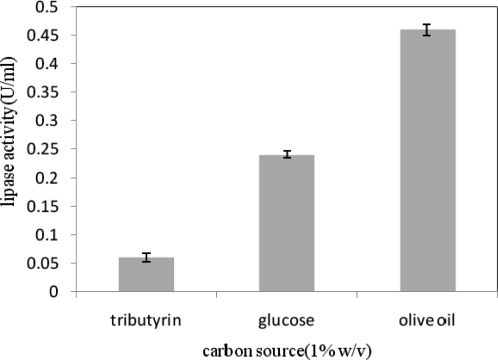
Effect ofdifferent carbon sources as additives(1% w/v) to the basal medium on lipase production by *Pseudomonas aeruginosa* KM110 (T: 30°C, pH: 7.0, agitation rate: 150 rpm).

**Effects of culture variable on lipase production**. The major factor for the expression of lipase activity has always been carbon, since lipases are inducible enzymes (10) and are thus generally produced in the presence of a lipid source such as oil or any other inducer, such as triacylglycerols, fatty acids, hydrolysable esters, tweens, bile salts and glycerol. However, their production is significantly influenced by other carbon sources such as sugars, polysaccharides, whey and other complex sources. Among the different carbon sources used, olive oil was found to be the most suitable source (Fig. 1). The maximum activity for olive oil was observed at 2% (v/v) (0.46U/ml). Most published experimental data have shown that lipid carbon sources (especially natural oils) stimulate lipase production (11–13). High levels of lipase production were reported from various thermophilic *Bacillus sp*. in the presence of olive oil as carbon source in the culture medium (14, 15). The presence of glucose in the cultivation medium depressed the production of lipase compared to olive oil. Glucose supplementation to the basal production medium inhibits lipase production, perhaps by catabolic repression. This occurred with reports for other lipase-producing organisms for which a high glucose concentration caused reduced lipase production (16–18).

**Fig. 2:**
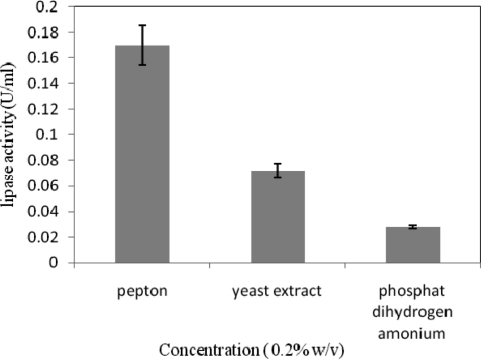
Effect of different nitrogen sources as additives (0.2% w/v) to the basal medium on lipase production by *Pseudomonas aeruginosa* KM110(T: 30°C, pH: 7.0, agitation rate: 150 rpm).

Beside carbon source, the type of nitrogen source in the medium also influenced the lipase titers in production broth. Generally, microorganisms provide high yields of lipase when organic nitrogen sources are used, such as peptone and yeast extract, which have been used for lipase production by various thermophilic *Bacillus sp*. and various *Pseudomonads* (19, 20). Among the different nitrogen sources used peptone was found to be the most suitable source (Fig. 2). In pepton (2 g/l) maximum activity was observed (0.17 U/ml).

**Fig. 3:**
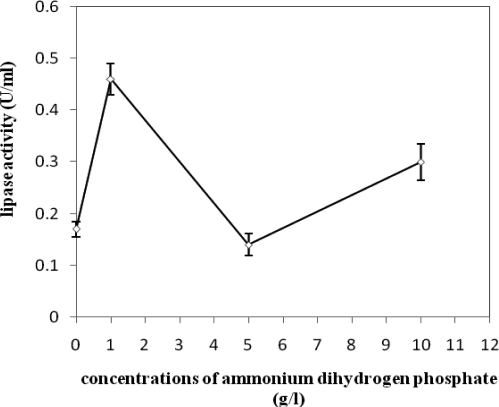
Effect of different concentrations of ammonium dihydrogen phosphate (g/l) on lipase production by *Pseudomonas aeruginosa* KM110 (T: 30°C, pH: 7.0, agitation rate: 150 rpm).

Inorganic nitrogen sources such as ammonium chloride and ammonium dihydrogen phosphate have been also reported to be effective in some microbes (21, 22). We also investigated the effect of ammonium dihydrogen phosphate as an inorganic carbon source with presence of peptone on lipase activity. Fig. 3 shows that it is effective on lipase activity so we used both peptone and ammonium dihydrogen phosphate as nitrogen sources in the medium.

**Fig. 4:**
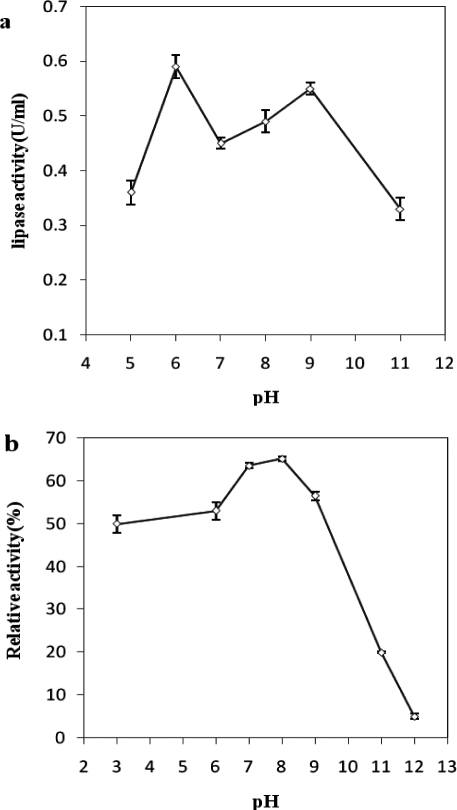
Effects of pH on activity (a) and stability (b) of lipase.

**Effect of pH on activity and stability of lipase**. The effect of pH on the activity of lipase was determined in four different buffers covering the range of pH 3.0 to 12.0. The enzyme was most active at pH 6.0 & 9.0 (Fig. 4a), increasing activity from pH 7-9 can be characterized as an alkalophilic enzyme but high lipase activity at pH 6 can be made the lipase applicable at acidic pH conditions. In general bacterial lipases are stable in a wide range of pH from 4 to 11. A comprehensive review of all bacterial lipase done by Gupta, et al. (4), states that maximum activity of lipases at pH values higher than 7 has been observed in many cases. Bacterial lipases have a neutral or alkaline optimum pH. with the exeption of lipase from *P. fluorescens* SIK W1 that has an acidic optimum pH 4.8. Also the lipase retained over 65% of its activity at pH 8.0 (Fig. 4b). Interestingly, other *Pseudomonas* lipases designated as alkaline, e.g., *P. fluorescens* HU380 (23), *P. mendocina* PK-12CS (24), *P. fluorescens* 2D (25) and *P. cepacia* (26) have lower pH optima of 8.5, 8.0, 8.5 and 9.0, respectively. Lipases from *P. pseudomalei* 12 sm (27) and *P. aeruginosa* YS-7 (28) both isolated from *Pseudomonas* growing in different water-restricted environments, are stable within the pH ranges of 7–10.5 and 6.5–7.5, respectively. Lipase obtained in our study was stable from pH 7.0 to 10.0. However, its stability was low at acidic pH. The remarkable stability of *Psudomonas aeruginosa* KM110 lipase in this range has proved it to be a potential alkaline lipase.

**Fig. 5:**
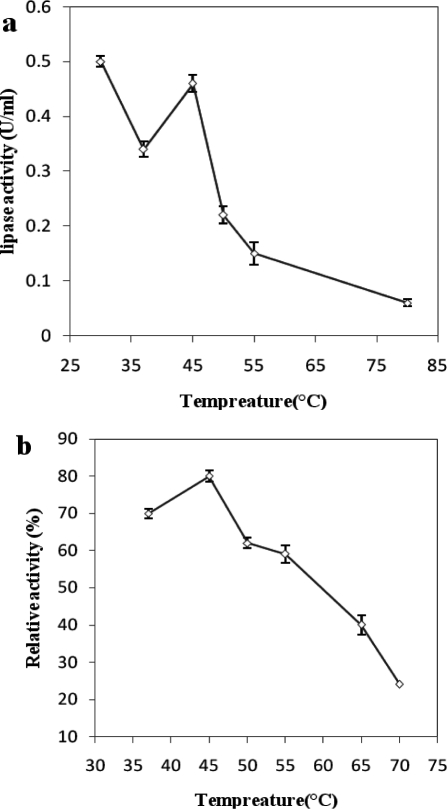
Effects of tempreature on activity (a) and stability (b) of lipase.

**Effect of temperature on activity & thermostabi-lity of lipase**. The temperature preference of this enzyme reveals higher activity values at temperature from 35 to 45°C (Fig. 5a). The lower activity at 35°C compared to 45°C, probably is due to the kinetics of enzymatic reaction as the enzyme is more conductive at 45°C. Assessment of the thermostability of lipase was performed by measuring the residual activity at various times, following incubation at different temperatures. As for the stability of the enzyme (Fig. 5b), 80% activity remained after 3 h of storage at 45°C and 70% at 37°C. At higher temperatures, the stability of the enzyme was lower; ie 40% activity remained after 3h at 65°C.The stability of the lipase decreased sharply after 1 h of incubation at high tempreatures (Fig. 5b). This indicates that *Psudomonas aeruginosa* KM110 lipase is a mesophilic enzyme. The optimum temperature of the lipase from *P. aeruginosa* EF2 was reported to be 50°C (29). The *P. aeruginosa* MB 5001 lipase has an optimum temperature of 55°C (30) but other *Pseudomonas* lipases, such as those from *P. fluorescens* 2D (23), *P. fluorescens* HU380 (23), *P. fragi* (31) and *P. mendoncina* (25) were found to be optimally active at 35–45°C*. P. aeruginosa* lipases seem to be more thermostable than others from this genus.

**The effect of metal ions on activity of lipase**. The effect of different metal ions on the activity of the lipase is shown in Table 1. Zn^**2+**^, Cu^**2+**^, and K^**+**^ salts decreased activity by 32, 27, and 21%, respectively, after 1 h of incubation at 30°C. Effect of the heavy metal ions Zn^**2+**^ and Cu^**2+**^ on the our lipase was similar to their effect on lipases from P.aeruginosa LP602 (17), P. *fluorescens* 2D (25) and HU380 (23). In contrast, activity increased around 40% in the presence of Fe^**2+**^ and 37% in the presence of Mg^**2+**^. It has been suggested that the effect of metal ions could be attributed to a change in the solubility and the behavior of the ionized fatty acids at interfaces, and from a change in the catalytic properties of the enzyme itself (32). This lipase was considered stable towards ions reported to inhibit other lipases (Fe^**2+**^ and Mg^**2+**^) (18, 32 and (33)). The metal ions Li^**+**^, Co^**2+**^, Mn^**2+**^ & Ca^**2+**^ showed a small inhibitory effect on lipase activity relative to Zn^**2+**^, Cu^**2+**^, and K^**+**^ salts and the lipase was relatively stable towards many other metal ions tested. This characteristic made the enzyme suitable for lipase reactions under various conditions without special precautions.

**Table 1:** Effects of various salts on lipase activity.

1 mM	Remaning activity (%)
control	100
FeCl2	139.5
MgCl2	136.8
NaCl	115.8
HgCl	115.8
NiCl	110.5
LiCl	92.1
CoCl	89.5
MnCl2	86.8
CaCl2	81.6
KCl	78.9
CuCl2	73.7
Zn(No3)2	68.4

**The effects of various detergent on activity of lipase**. Table 2 demonstrates the effects of various detergents on enzyme activity. *P. aeruginosa* KM110 lipase was sensitive to SDS than the other. In 1.0% SDS concentration, the enzymes showed 70% activity after 1 h storage. This level of stability was similar to or higher than those reported for other bacteria (32).The enzyme was relatively stable when stored with Tween-80, DMSO and Triton X-100 (i.e., the activity remaining higher than 80%). In accordance to our results, Schmidt- Dannert et al. 1994 (34) reported a total loss of lipolytic activity in the presence of Tween 20 and Tween 80, but no effect was observed when incubated with Triton X-100. Nawani et al. (1998) also found a total loss of activity in the presence of SDS but in contrast, activity was enhanced in the presence of Triton X-100, Tween 20 and Tween 80.

**Table 2:** Effects of various detergents on lipase activity.

1% concentration	Remaining activity(%)
control	100
DMSO	102.8
Triton X-100	91.4
Tween 80	82.8
SDS	71.4

## CONCLUSION

The results obtained in this study show that olive oil and peptone were the most suitable substrate for maximum lipase production by *P. aeruginosa* KM110**. Further studies are needed to enhance lipase production in this strain. We are currently in the process of cloning this lipase and placing it under a strong promoter to be able to determine molecular properties of the lipase as well as increasing enzyme expression and yields for future industrial applications. Also, *P. aeruginosa* KM110 lipase is a potential alkaline lipase. The enzyme exhibited maximum activity and stability between pH 7.0 and 10.0, but the stability was low at acidic pH. The remarkable stability of *P. aeruginosa* KM110 lipase in this range has proved it to be a potential alkaline lipase similar to other, and a candidate for industrial applications such as detergent, leather and fine chemical industries. Also, the lipase was relatively stable towards many metal ions and detergents tested. This characteristic made the enzyme suitable for lipase reactions under various conditions without special precautions.
